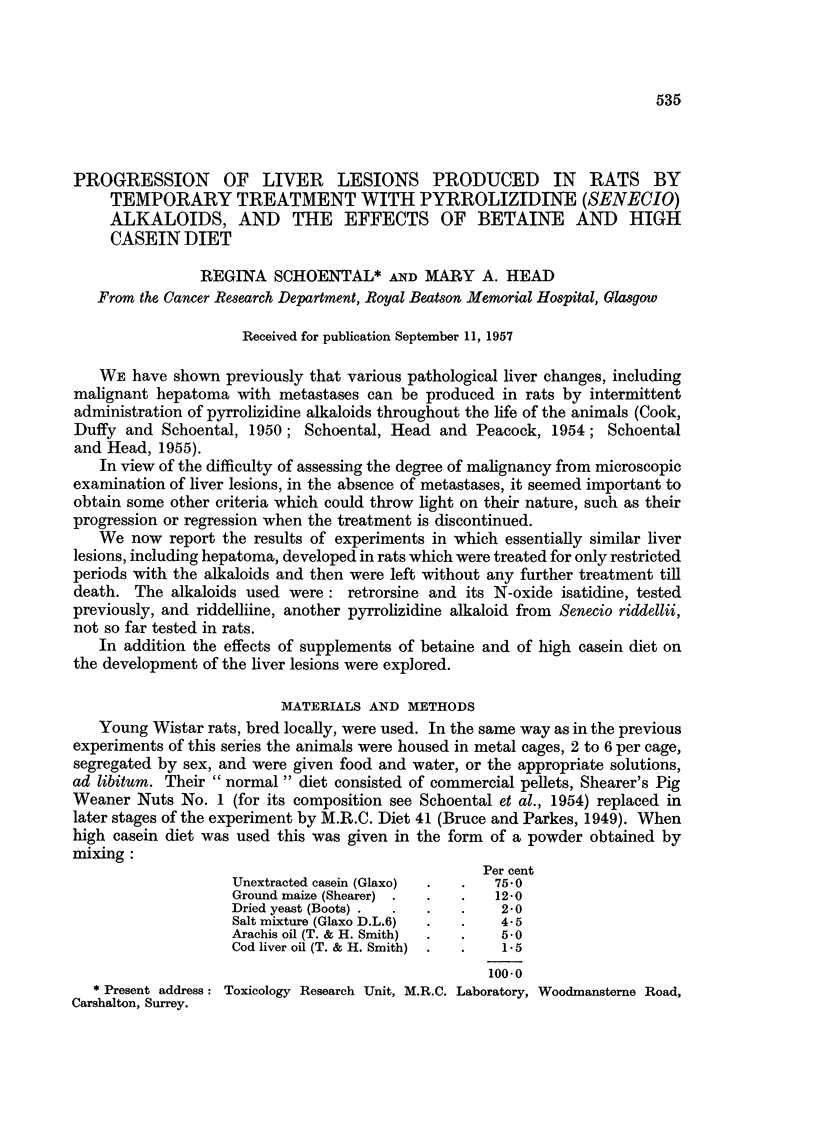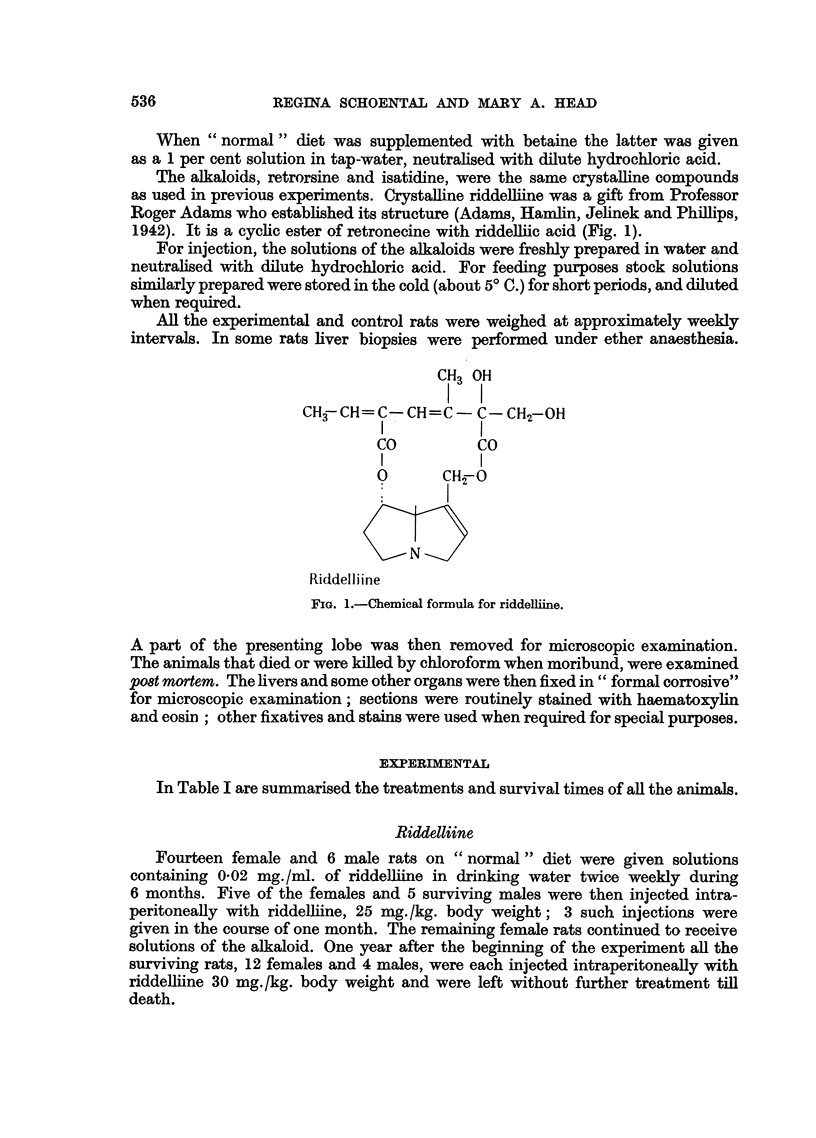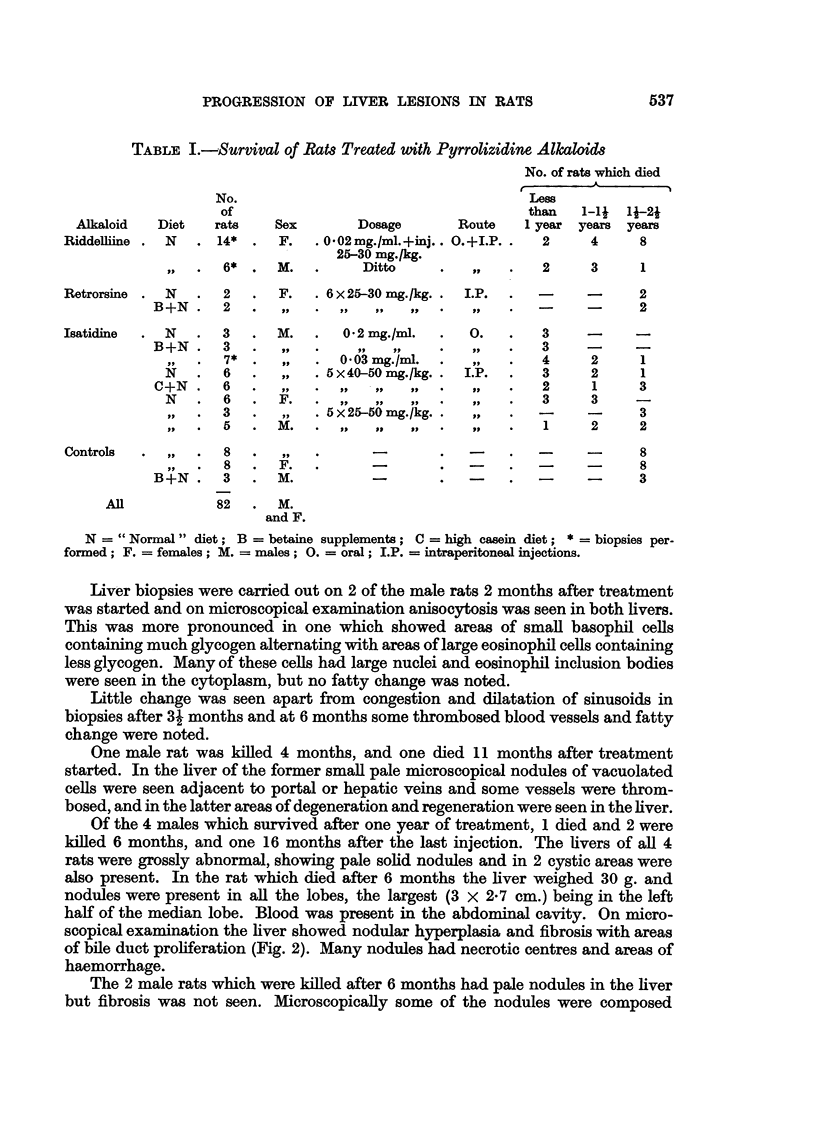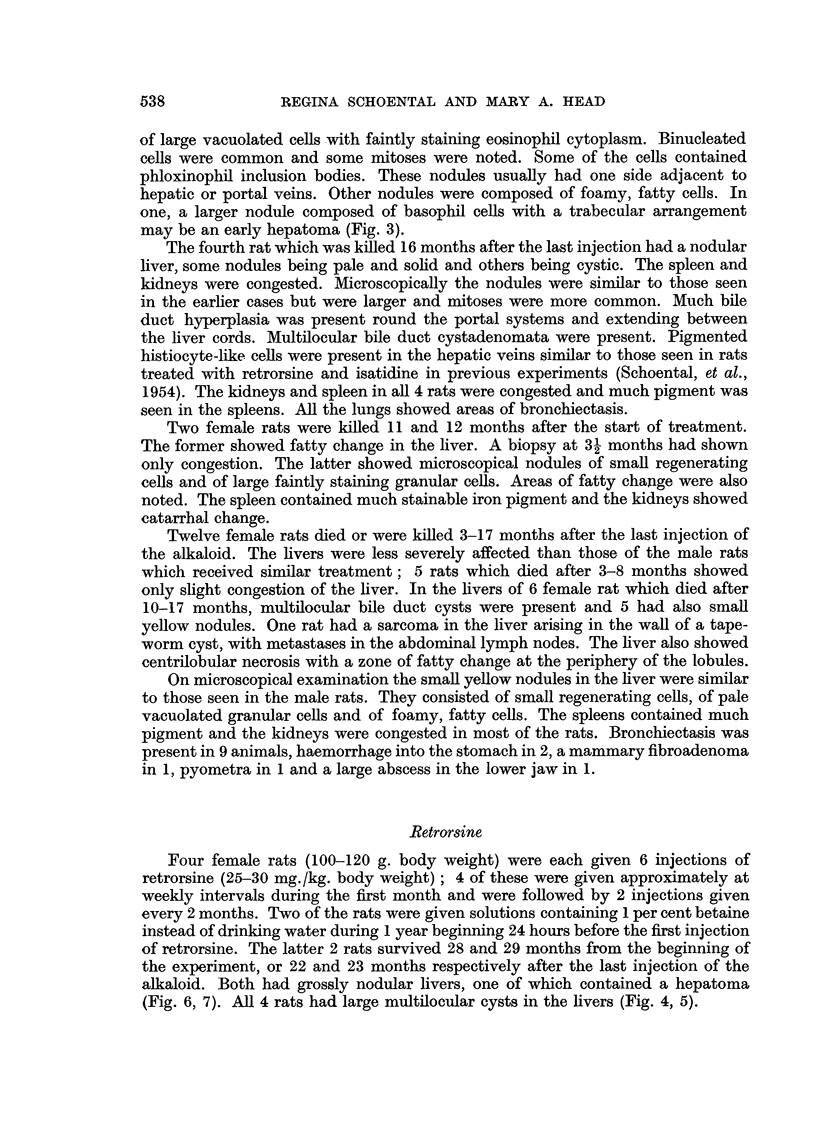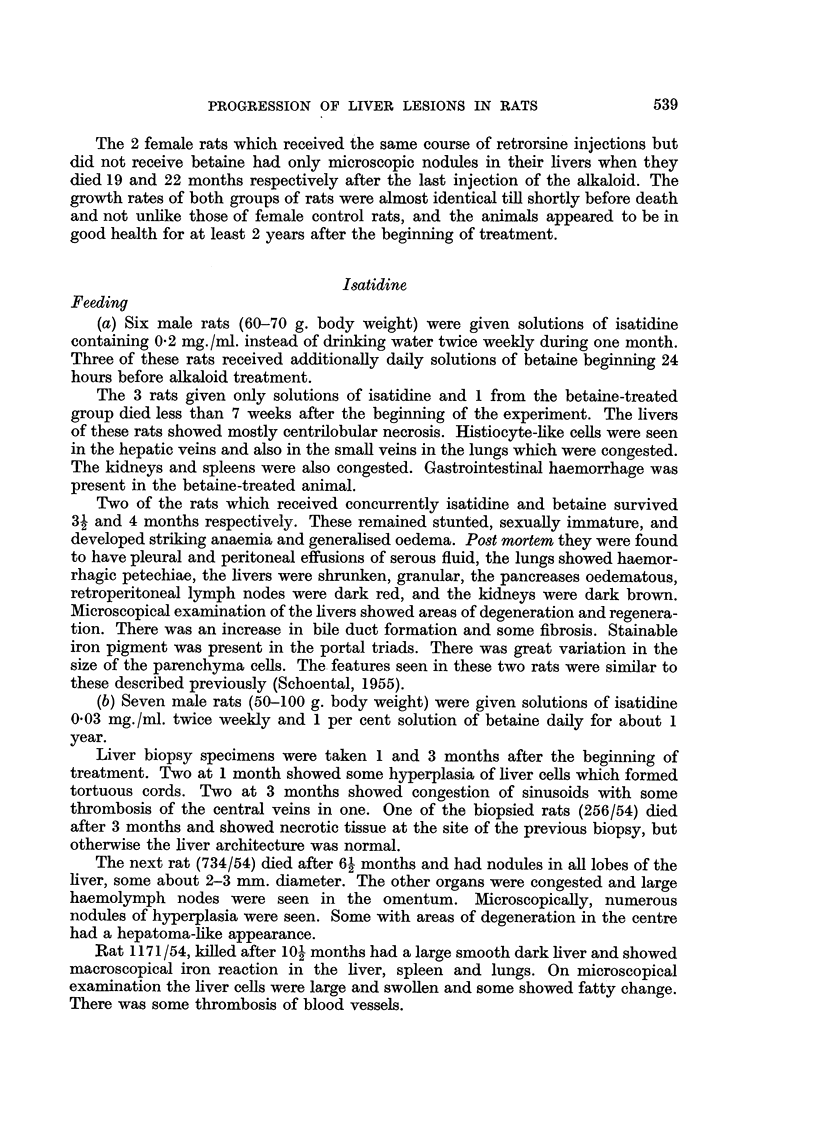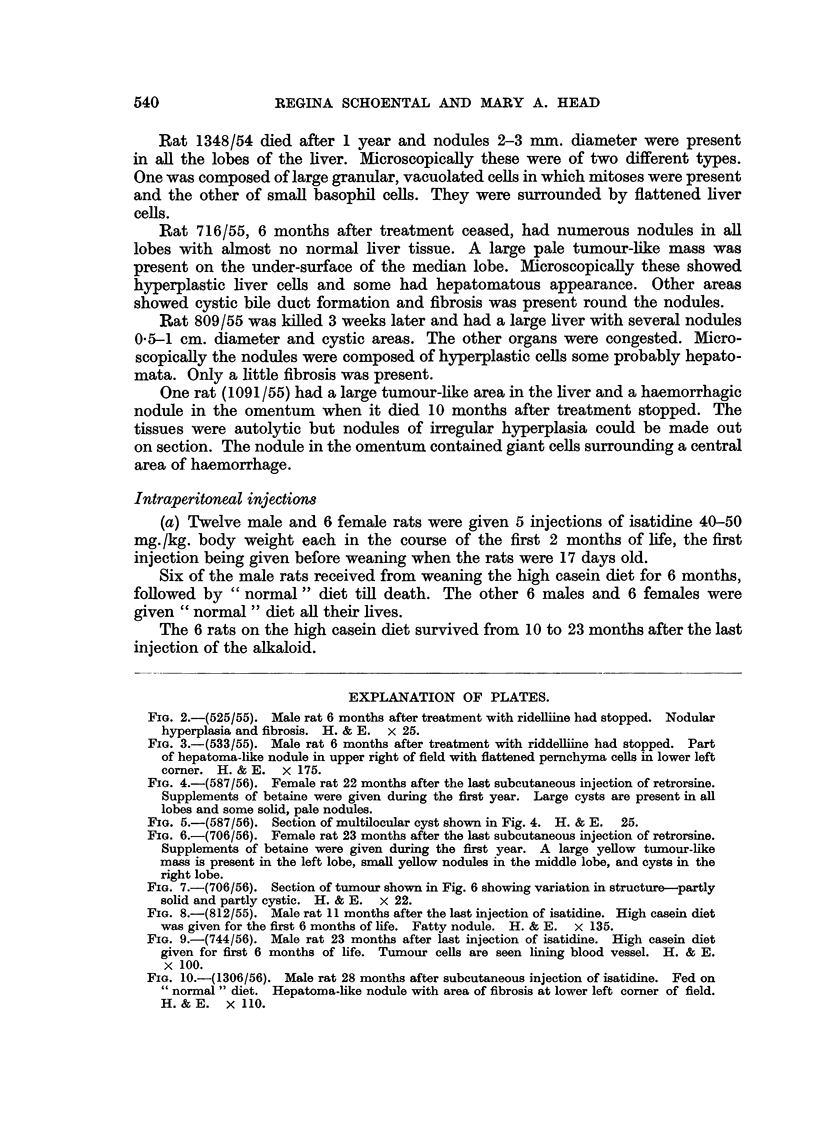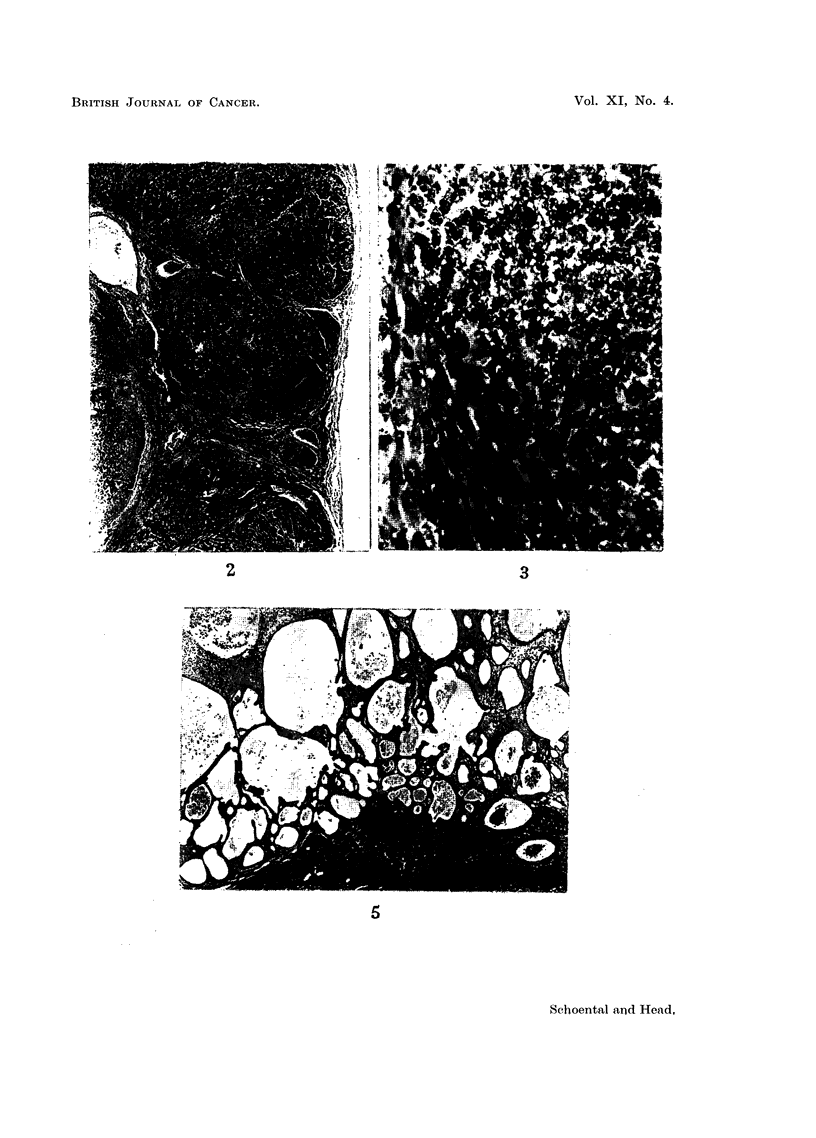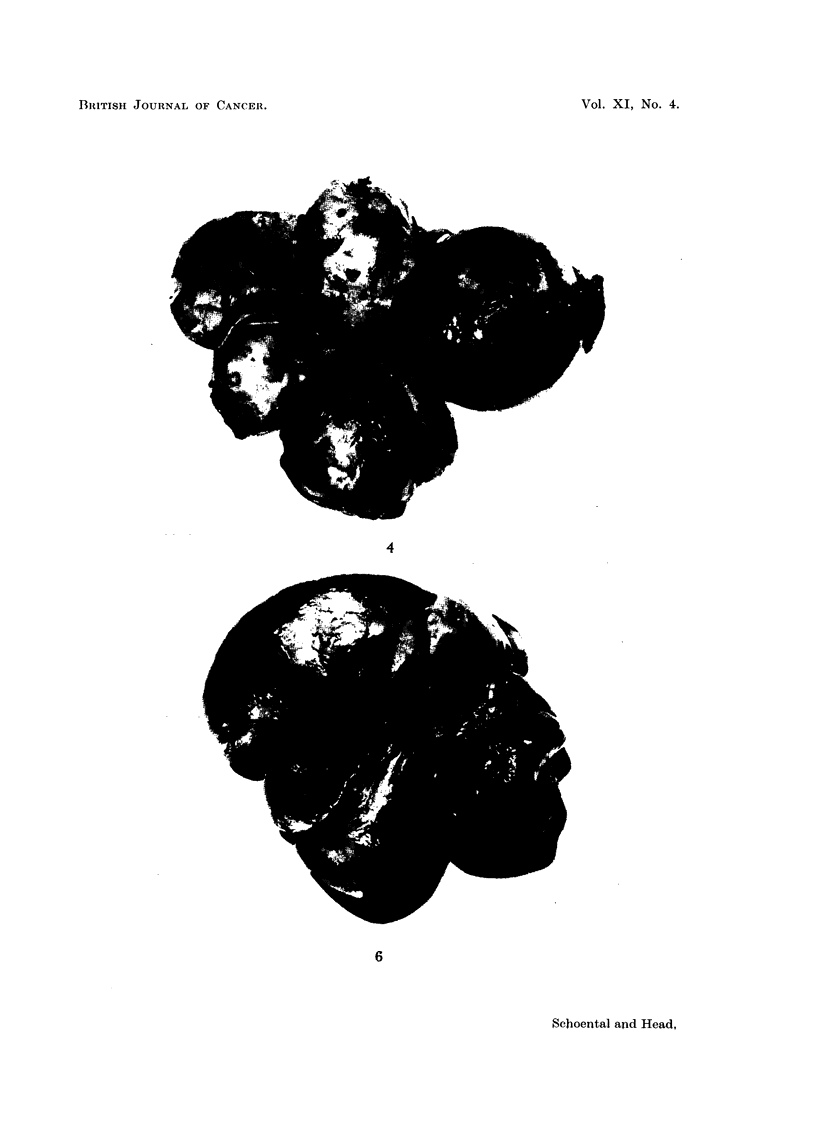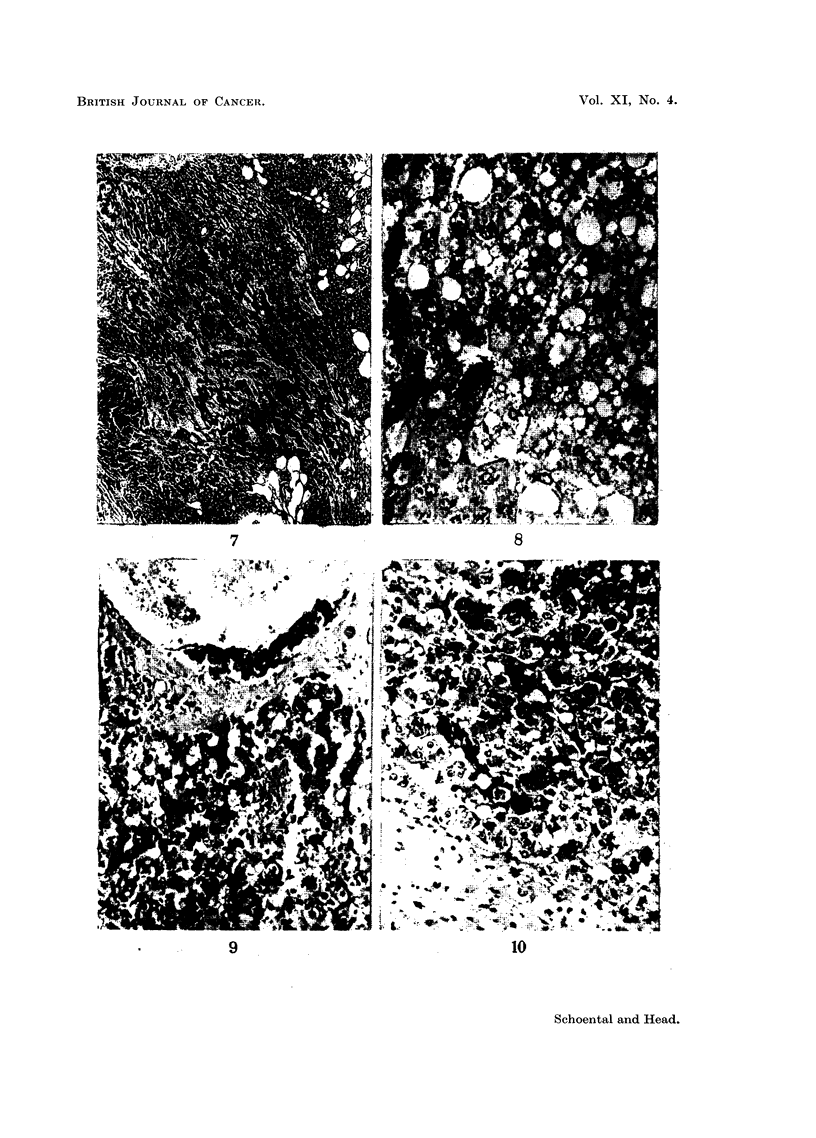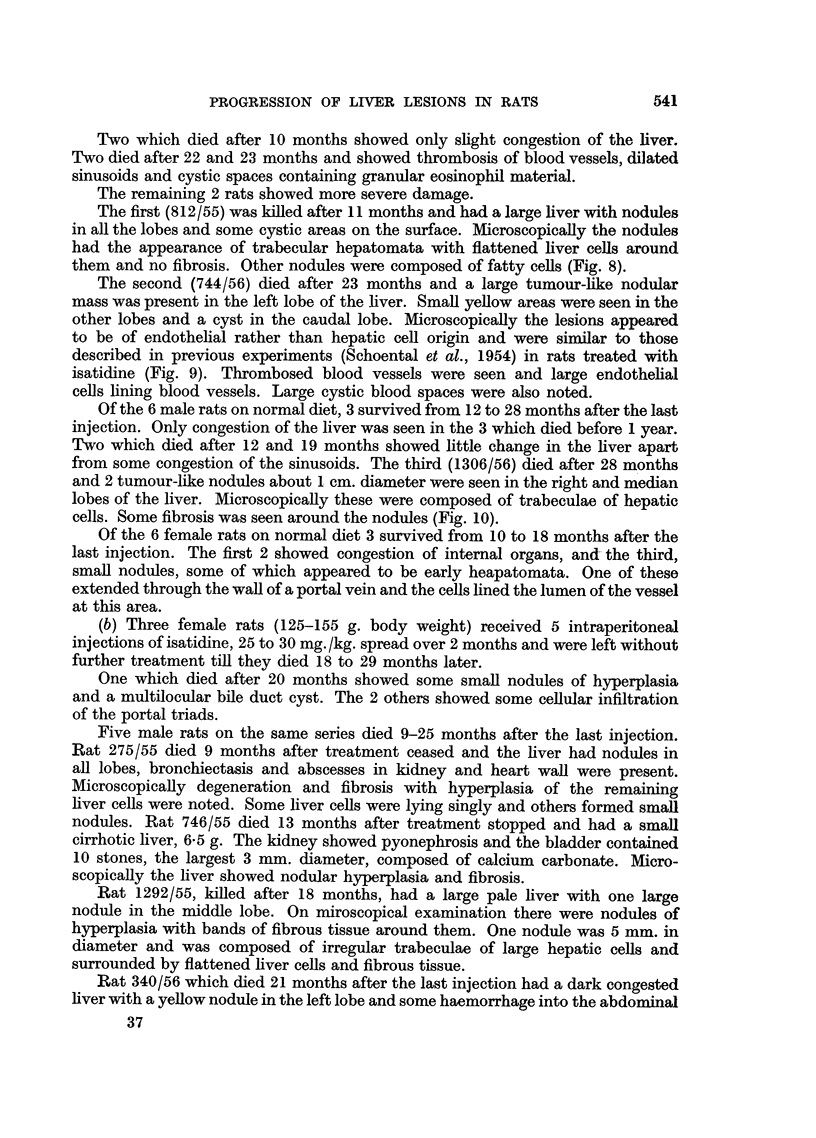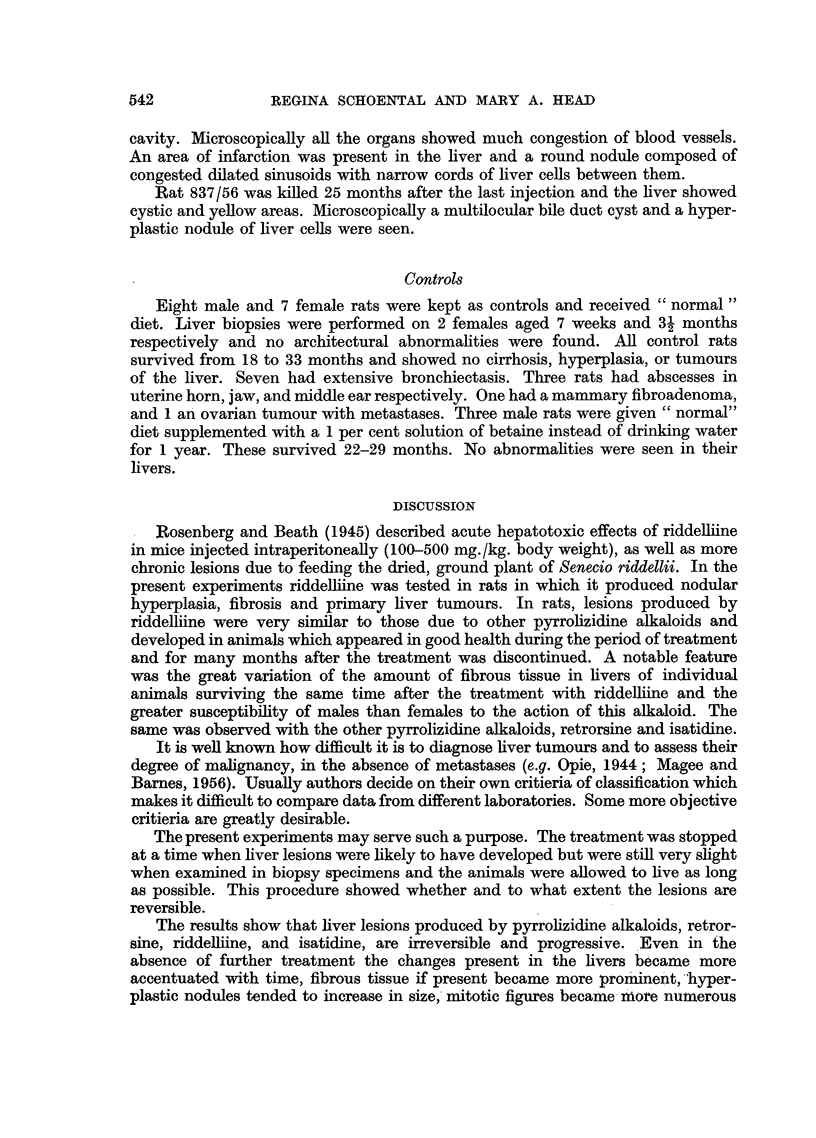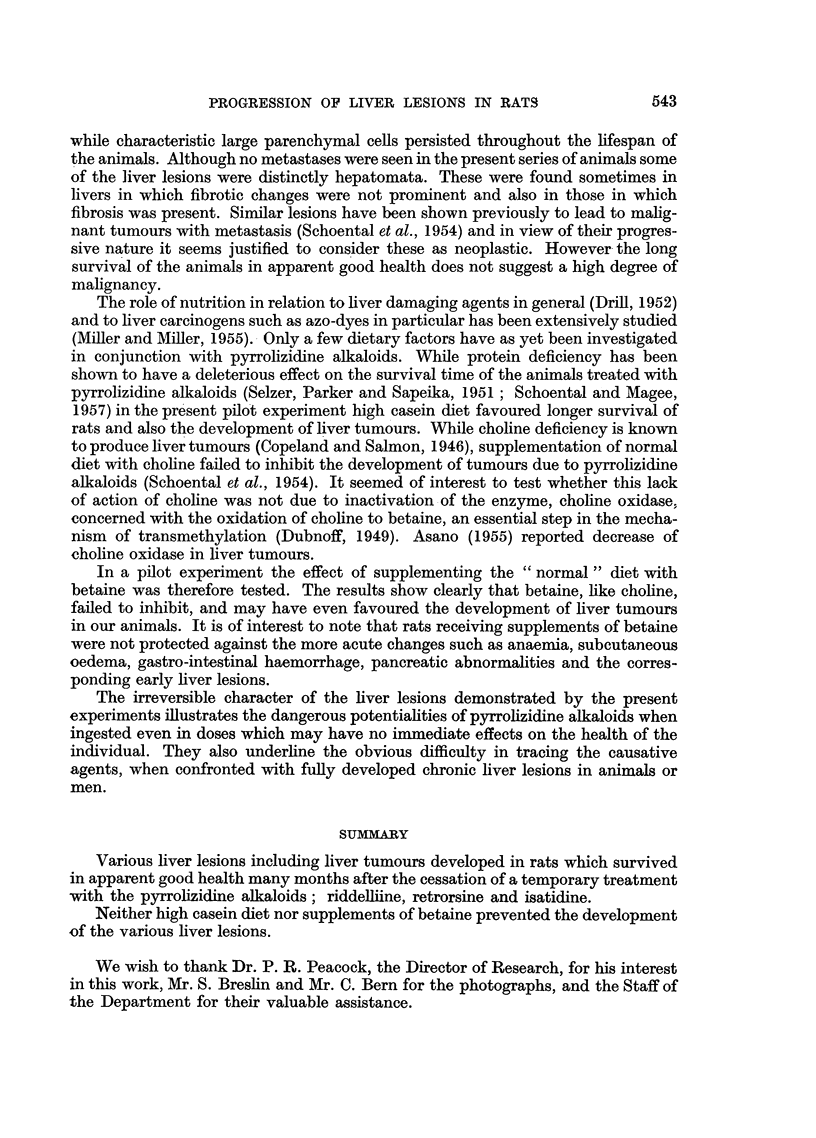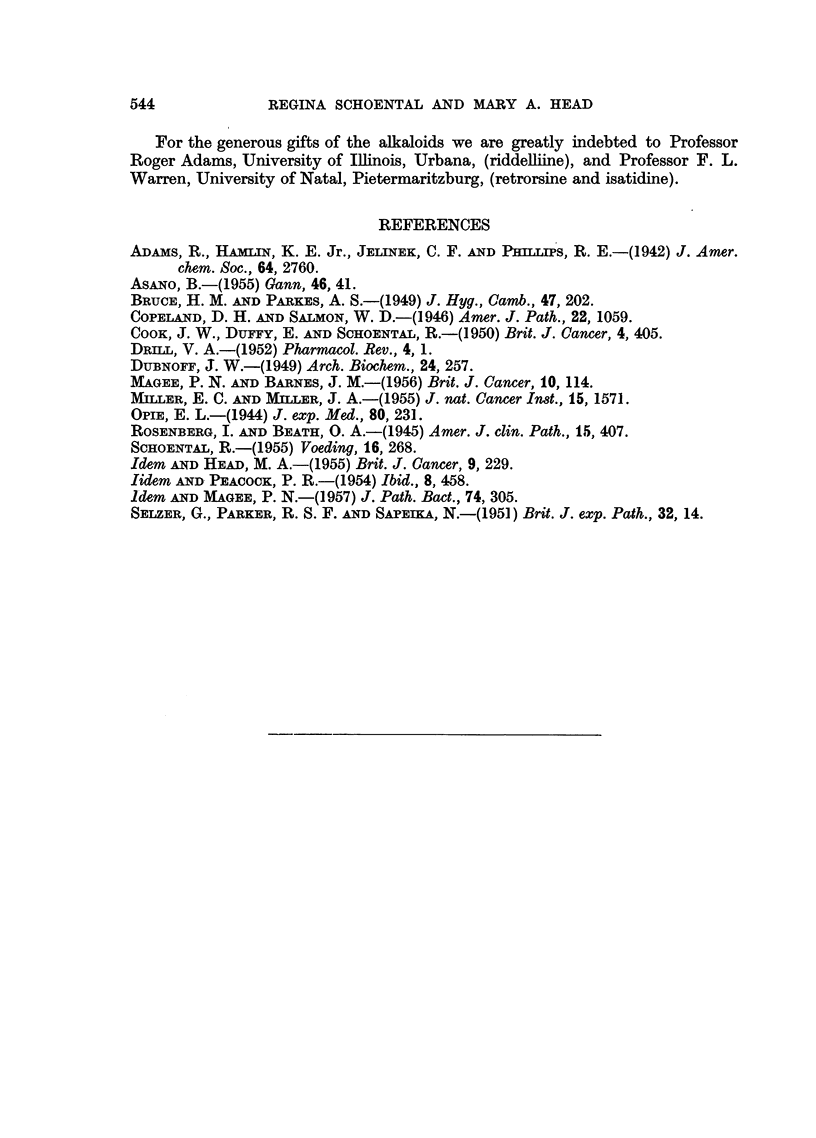# Progression of Liver Lesions Produced in Rats by Temporary Treatment with Pyrrolizidine (Senecio) Alkaloids, and the Effects of Betaine and High Casein Diet

**DOI:** 10.1038/bjc.1957.65

**Published:** 1957-12

**Authors:** Regina Schoental, Mary A. Head

## Abstract

**Images:**


### PROGRESSION OF LIVER LESIONS PRODUCED IN RATS BY TEMPORARY TREATMENT WITH PYRROLIZIDINE (*SENECIO*) ALKALOIDS, AND THE EFFECTS OF BETAINE AND HIGH CASEIN DIET

REGINA SCHOENTAL* AND MARY A. HEAD

*From the Cancer Research Department, Royal Beatson Memorial Hospital, Glasgow*

Received for publication September 11, 1957

WE have shown previously that various pathological liver changes, including malignant hepatoma with metastases can be produced in rats by intermittent administration of pyrrolizidine alkaloids throughout the life of the animals (Cook, Duffy and Schoental, 1950; Schoental, Head and Peacock, 1954; Schoental and Head, 1955).

In view of the difficulty of assessing the degree of malignancy from microscopic examination of liver lesions, in the absence of metastases, it seemed important to obtain some other criteria which could throw light on their nature, such as their progression or regression when the treatment is discontinued.

We now report the results of experiments in which essentially similar liver lesions, including hepatoma, developed in rats which were treated for only restricted periods with the alkaloids and then were left without any further treatment till death. The alkaloids used were: retrorsine and its N-oxide isatidine, tested previously, and riddelliine, another pyrrolizidine alkaloid from *Senecio riddellii*, not so far tested in rats.

In addition the effects of supplements of betaine and of high casein diet on the development of the liver lesions were explored.

### MATERIALS AND METHODS

Young Wistar rats, bred locally, were used. In the same way as in the previous experiments of this series the animals were housed in metal cages, 2 to 6 per cage, segregated by sex, and were given food and water, or the appropriate solutions, *ad libitum*. Their " normal " diet consisted of commercial pellets, Shearer's Pig Weaner Nuts No. 1 (for its composition see Schoental *et al.*, 1954) replaced in later stages of the experiment by M.R.C. Diet 41 (Bruce and Parkes, 1949). When high casein diet was used this was given in the form of a powder obtained by mixing:

| | Per cent |
|---|---|
| Unextracted casein (Glaxo) | 75·0 |
| Ground maize (Shearer) | 12·0 |
| Dried yeast (Boots) | 2·0 |
| Salt mixture (Glaxo D.L.6) | 4·5 |
| Arachis oil (T. & H. Smith) | 5·0 |
| Cod liver oil (T. & H. Smith) | 1·5 |
| | 100·0 |

* Present address: Toxicology Research Unit, M.R.C. Laboratory, Woodmansterne Road, Carshalton, Surrey.

When " normal " diet was supplemented with betaine the latter was given as a 1 per cent solution in tap-water, neutralised with dilute hydrochloric acid.

The alkaloids, retrorsine and isatidine, were the same crystalline compounds as used in previous experiments. Crystalline riddelliine was a gift from Professor Roger Adams who established its structure (Adams, Hamlin, Jelinek and Phillips, 1942). It is a cyclic ester of retronecine with riddelliic acid (Fig. 1).

For injection, the solutions of the alkaloids were freshly prepared in water and neutralised with dilute hydrochloric acid. For feeding purposes stock solutions similarly prepared were stored in the cold (about 5° C.) for short periods, and diluted when required.

All the experimental and control rats were weighed at approximately weekly intervals. In some rats liver biopsies were performed under ether anaesthesia.

Riddelliine

FIG. 1.—Chemical formula for riddelliine.

A part of the presenting lobe was then removed for microscopic examination. The animals that died or were killed by chloroform when moribund, were examined *post mortem*. The livers and some other organs were then fixed in " formal corrosive" for microscopic examination ; sections were routinely stained with haematoxylin and eosin ; other fixatives and stains were used when required for special purposes.

### EXPERIMENTAL

In Table I are summarised the treatments and survival times of all the animals.

### *Riddelliine*

Fourteen female and 6 male rats on " normal " diet were given solutions containing 0·02 mg./ml. of riddelliine in drinking water twice weekly during 6 months. Five of the females and 5 surviving males were then injected intraperitoneally with riddelliine, 25 mg./kg. body weight ; 3 such injections were given in the course of one month. The remaining female rats continued to receive solutions of the alkaloid. One year after the beginning of the experiment all the surviving rats, 12 females and 4 males, were each injected intraperitoneally with riddelliine 30 mg./kg. body weight and were left without further treatment till death.

TABLE I.—*Survival of Rats Treated with Pyrrolizidine Alkaloids*

| Alkaloid | Diet | No. of rats | Sex | Dosage | Route | No. of rats which died: Less than 1 year | 1–1½ years | 1½–2½ years |
|---|---|---|---|---|---|---|---|---|
| Riddelliine | N | 14* | F. | 0·02 mg./ml. + inj. 25–30 mg./kg. | O. + I.P. | 2 | 4 | 8 |
| | ,, | 6* | M. | Ditto | ,, | 2 | 3 | 1 |
| Retrorsine | N | 2 | F. | 6 × 25–30 mg./kg. | I.P. | — | — | 2 |
| | B+N | 2 | ,, | ,, ,, ,, | ,, | — | — | 2 |
| Isatidine | N | 3 | M. | 0·2 mg./ml. | O. | 3 | — | — |
| | B+N | 3 | ,, | ,, ,, | ,, | 3 | — | — |
| | ,, | 7* | ,, | 0·03 mg./ml. | ,, | 4 | 2 | 1 |
| | N | 6 | ,, | 5 × 40–50 mg./kg. | I.P. | 3 | 2 | 1 |
| | C+N | 6 | ,, | ,, ,, ,, | ,, | 2 | 1 | 3 |
| | N | 6 | F. | ,, ,, ,, | ,, | 3 | 3 | — |
| | ,, | 3 | ,, | 5 × 25–50 mg./kg. | ,, | — | — | 3 |
| | ,, | 5 | M. | ,, ,, ,, | ,, | 1 | 2 | 2 |
| Controls | ,, | 8 | ,, | — | — | — | — | 8 |
| | ,, | 8 | F. | — | — | — | — | 8 |
| | B+N | 3 | M. | — | — | — | — | 3 |
| All | | 82 | M. and F. | | | | | |

N = " Normal " diet ; B = betaine supplements ; C = high casein diet ; * = biopsies performed ; F. = females ; M. = males ; O. = oral ; I.P. = intraperitoneal injections.

Liver biopsies were carried out on 2 of the male rats 2 months after treatment was started and on microscopical examination anisocytosis was seen in both livers. This was more pronounced in one which showed areas of small basophil cells containing much glycogen alternating with areas of large eosinophil cells containing less glycogen. Many of these cells had large nuclei and eosinophil inclusion bodies were seen in the cytoplasm, but no fatty change was noted.

Little change was seen apart from congestion and dilatation of sinusoids in biopsies after 3½ months and at 6 months some thrombosed blood vessels and fatty change were noted.

One male rat was killed 4 months, and one died 11 months after treatment started. In the liver of the former small pale microscopical nodules of vacuolated cells were seen adjacent to portal or hepatic veins and some vessels were thrombosed, and in the latter areas of degeneration and regeneration were seen in the liver.

Of the 4 males which survived after one year of treatment, 1 died and 2 were killed 6 months, and one 16 months after the last injection. The livers of all 4 rats were grossly abnormal, showing pale solid nodules and in 2 cystic areas were also present. In the rat which died after 6 months the liver weighed 30 g. and nodules were present in all the lobes, the largest (3 × 2·7 cm.) being in the left half of the median lobe. Blood was present in the abdominal cavity. On microscopical examination the liver showed nodular hyperplasia and fibrosis with areas of bile duct proliferation (Fig. 2). Many nodules had necrotic centres and areas of haemorrhage.

The 2 male rats which were killed after 6 months had pale nodules in the liver but fibrosis was not seen. Microscopically some of the nodules were composed of large vacuolated cells with faintly staining eosinophil cytoplasm. Binucleated cells were common and some mitoses were noted. Some of the cells contained phloxinophil inclusion bodies. These nodules usually had one side adjacent to hepatic or portal veins. Other nodules were composed of foamy, fatty cells. In one, a larger nodule composed of basophil cells with a trabecular arrangement may be an early hepatoma (Fig. 3).

The fourth rat which was killed 16 months after the last injection had a nodular liver, some nodules being pale and solid and others being cystic. The spleen and kidneys were congested. Microscopically the nodules were similar to those seen in the earlier cases but were larger and mitoses were more common. Much bile duct hyperplasia was present round the portal systems and extending between the liver cords. Multilocular bile duct cystadenomata were present. Pigmented histiocyte-like cells were present in the hepatic veins similar to those seen in rats treated with retrorsine and isatidine in previous experiments (Schoental, *et al.*, 1954). The kidneys and spleen in all 4 rats were congested and much pigment was seen in the spleens. All the lungs showed areas of bronchiectasis.

Two female rats were killed 11 and 12 months after the start of treatment. The former showed fatty change in the liver. A biopsy at $3\frac{1}{2}$ months had shown only congestion. The latter showed microscopical nodules of small regenerating cells and of large faintly staining granular cells. Areas of fatty change were also noted. The spleen contained much stainable iron pigment and the kidneys showed catarrhal change.

Twelve female rats died or were killed 3–17 months after the last injection of the alkaloid. The livers were less severely affected than those of the male rats which received similar treatment ; 5 rats which died after 3–8 months showed only slight congestion of the liver. In the livers of 6 female rat which died after 10–17 months, multilocular bile duct cysts were present and 5 had also small yellow nodules. One rat had a sarcoma in the liver arising in the wall of a tapeworm cyst, with metastases in the abdominal lymph nodes. The liver also showed centrilobular necrosis with a zone of fatty change at the periphery of the lobules.

On microscopical examination the small yellow nodules in the liver were similar to those seen in the male rats. They consisted of small regenerating cells, of pale vacuolated granular cells and of foamy, fatty cells. The spleens contained much pigment and the kidneys were congested in most of the rats. Bronchiectasis was present in 9 animals, haemorrhage into the stomach in 2, a mammary fibroadenoma in 1, pyometra in 1 and a large abscess in the lower jaw in 1.

### *Retrorsine*

Four female rats (100–120 g. body weight) were each given 6 injections of retrorsine (25–30 mg./kg. body weight) ; 4 of these were given approximately at weekly intervals during the first month and were followed by 2 injections given every 2 months. Two of the rats were given solutions containing 1 per cent betaine instead of drinking water during 1 year beginning 24 hours before the first injection of retrorsine. The latter 2 rats survived 28 and 29 months from the beginning of the experiment, or 22 and 23 months respectively after the last injection of the alkaloid. Both had grossly nodular livers, one of which contained a hepatoma (Fig. 6, 7). All 4 rats had large multilocular cysts in the livers (Fig. 4, 5).

The 2 female rats which received the same course of retrorsine injections but did not receive betaine had only microscopic nodules in their livers when they died 19 and 22 months respectively after the last injection of the alkaloid. The growth rates of both groups of rats were almost identical till shortly before death and not unlike those of female control rats, and the animals appeared to be in good health for at least 2 years after the beginning of treatment.

### *Isatidine*

*Feeding*

(*a*) Six male rats (60–70 g. body weight) were given solutions of isatidine containing 0·2 mg./ml. instead of drinking water twice weekly during one month. Three of these rats received additionally daily solutions of betaine beginning 24 hours before alkaloid treatment.

The 3 rats given only solutions of isatidine and 1 from the betaine-treated group died less than 7 weeks after the beginning of the experiment. The livers of these rats showed mostly centrilobular necrosis. Histiocyte-like cells were seen in the hepatic veins and also in the small veins in the lungs which were congested. The kidneys and spleens were also congested. Gastrointestinal haemorrhage was present in the betaine-treated animal.

Two of the rats which received concurrently isatidine and betaine survived $3\frac{1}{2}$ and 4 months respectively. These remained stunted, sexually immature, and developed striking anaemia and generalised oedema. *Post mortem* they were found to have pleural and peritoneal effusions of serous fluid, the lungs showed haemorrhagic petechiae, the livers were shrunken, granular, the pancreases oedematous, retroperitoneal lymph nodes were dark red, and the kidneys were dark brown. Microscopical examination of the livers showed areas of degeneration and regeneration. There was an increase in bile duct formation and some fibrosis. Stainable iron pigment was present in the portal triads. There was great variation in the size of the parenchyma cells. The features seen in these two rats were similar to these described previously (Schoental, 1955).

(*b*) Seven male rats (50–100 g. body weight) were given solutions of isatidine 0·03 mg./ml. twice weekly and 1 per cent solution of betaine daily for about 1 year.

Liver biopsy specimens were taken 1 and 3 months after the beginning of treatment. Two at 1 month showed some hyperplasia of liver cells which formed tortuous cords. Two at 3 months showed congestion of sinusoids with some thrombosis of the central veins in one. One of the biopsied rats (256/54) died after 3 months and showed necrotic tissue at the site of the previous biopsy, but otherwise the liver architecture was normal.

The next rat (734/54) died after $6\frac{1}{2}$ months and had nodules in all lobes of the liver, some about 2–3 mm. diameter. The other organs were congested and large haemolymph nodes were seen in the omentum. Microscopically, numerous nodules of hyperplasia were seen. Some with areas of degeneration in the centre had a hepatoma-like appearance.

Rat 1171/54, killed after $10\frac{1}{2}$ months had a large smooth dark liver and showed macroscopical iron reaction in the liver, spleen and lungs. On microscopical examination the liver cells were large and swollen and some showed fatty change. There was some thrombosis of blood vessels.

Rat 1348/54 died after 1 year and nodules 2–3 mm. diameter were present in all the lobes of the liver. Microscopically these were of two different types. One was composed of large granular, vacuolated cells in which mitoses were present and the other of small basophil cells. They were surrounded by flattened liver cells.

Rat 716/55, 6 months after treatment ceased, had numerous nodules in all lobes with almost no normal liver tissue. A large pale tumour-like mass was present on the under-surface of the median lobe. Microscopically these showed hyperplastic liver cells and some had hepatomatous appearance. Other areas showed cystic bile duct formation and fibrosis was present round the nodules.

Rat 809/55 was killed 3 weeks later and had a large liver with several nodules 0·5–1 cm. diameter and cystic areas. The other organs were congested. Microscopically the nodules were composed of hyperplastic cells some probably hepatomata. Only a little fibrosis was present.

One rat (1091/55) had a large tumour-like area in the liver and a haemorrhagic nodule in the omentum when it died 10 months after treatment stopped. The tissues were autolytic but nodules of irregular hyperplasia could be made out on section. The nodule in the omentum contained giant cells surrounding a central area of haemorrhage.

*Intraperitoneal injections*

(*a*) Twelve male and 6 female rats were given 5 injections of isatidine 40–50 mg./kg. body weight each in the course of the first 2 months of life, the first injection being given before weaning when the rats were 17 days old.

Six of the male rats received from weaning the high casein diet for 6 months, followed by " normal " diet till death. The other 6 males and 6 females were given " normal " diet all their lives.

The 6 rats on the high casein diet survived from 10 to 23 months after the last injection of the alkaloid.

---

EXPLANATION OF PLATES.

FIG. 2.—(525/55). Male rat 6 months after treatment with ridelliine had stopped. Nodular hyperplasia and fibrosis. H. & E. × 25.

FIG. 3.—(533/55). Male rat 6 months after treatment with riddelliine had stopped. Part of hepatoma-like nodule in upper right of field with flattened pernchyma cells in lower left corner. H. & E. × 175.

FIG. 4.—(587/56). Female rat 22 months after the last subcutaneous injection of retrorsine. Supplements of betaine were given during the first year. Large cysts are present in all lobes and some solid, pale nodules.

FIG. 5.—(587/56). Section of multilocular cyst shown in Fig. 4. H. & E. 25.

FIG. 6.—(706/56). Female rat 23 months after the last subcutaneous injection of retrorsine. Supplements of betaine were given during the first year. A large yellow tumour-like mass is present in the left lobe, small yellow nodules in the middle lobe, and cysts in the right lobe.

FIG. 7.—(706/56). Section of tumour shown in Fig. 6 showing variation in structure—partly solid and partly cystic. H. & E. × 22.

FIG. 8.—(812/55). Male rat 11 months after the last injection of isatidine. High casein diet was given for the first 6 months of life. Fatty nodule. H. & E. × 135.

FIG. 9.—(744/56). Male rat 23 months after last injection of isatidine. High casein diet given for first 6 months of life. Tumour cells are seen lining blood vessel. H. & E. × 100.

FIG. 10.—(1306/56). Male rat 28 months after subcutaneous injection of isatidine. Fed on " normal " diet. Hepatoma-like nodule with area of fibrosis at lower left corner of field. H. & E. × 110.

2

3

5

4

6

Schoental and Head.

7

8

9

10

Two which died after 10 months showed only slight congestion of the liver. Two died after 22 and 23 months and showed thrombosis of blood vessels, dilated sinusoids and cystic spaces containing granular eosinophil material.

The remaining 2 rats showed more severe damage.

The first (812/55) was killed after 11 months and had a large liver with nodules in all the lobes and some cystic areas on the surface. Microscopically the nodules had the appearance of trabecular hepatomata with flattened liver cells around them and no fibrosis. Other nodules were composed of fatty cells (Fig. 8).

The second (744/56) died after 23 months and a large tumour-like nodular mass was present in the left lobe of the liver. Small yellow areas were seen in the other lobes and a cyst in the caudal lobe. Microscopically the lesions appeared to be of endothelial rather than hepatic cell origin and were similar to those described in previous experiments (Schoental *et al.*, 1954) in rats treated with isatidine (Fig. 9). Thrombosed blood vessels were seen and large endothelial cells lining blood vessels. Large cystic blood spaces were also noted.

Of the 6 male rats on normal diet, 3 survived from 12 to 28 months after the last injection. Only congestion of the liver was seen in the 3 which died before 1 year. Two which died after 12 and 19 months showed little change in the liver apart from some congestion of the sinusoids. The third (1306/56) died after 28 months and 2 tumour-like nodules about 1 cm. diameter were seen in the right and median lobes of the liver. Microscopically these were composed of trabeculae of hepatic cells. Some fibrosis was seen around the nodules (Fig. 10).

Of the 6 female rats on normal diet 3 survived from 10 to 18 months after the last injection. The first 2 showed congestion of internal organs, and the third, small nodules, some of which appeared to be early heapatomata. One of these extended through the wall of a portal vein and the cells lined the lumen of the vessel at this area.

(*b*) Three female rats (125–155 g. body weight) received 5 intraperitoneal injections of isatidine, 25 to 30 mg./kg. spread over 2 months and were left without further treatment till they died 18 to 29 months later.

One which died after 20 months showed some small nodules of hyperplasia and a multilocular bile duct cyst. The 2 others showed some cellular infiltration of the portal triads.

Five male rats on the same series died 9–25 months after the last injection. Rat 275/55 died 9 months after treatment ceased and the liver had nodules in all lobes, bronchiectasis and abscesses in kidney and heart wall were present. Microscopically degeneration and fibrosis with hyperplasia of the remaining liver cells were noted. Some liver cells were lying singly and others formed small nodules. Rat 746/55 died 13 months after treatment stopped and had a small cirrhotic liver, 6·5 g. The kidney showed pyonephrosis and the bladder contained 10 stones, the largest 3 mm. diameter, composed of calcium carbonate. Microscopically the liver showed nodular hyperplasia and fibrosis.

Rat 1292/55, killed after 18 months, had a large pale liver with one large nodule in the middle lobe. On miroscopical examination there were nodules of hyperplasia with bands of fibrous tissue around them. One nodule was 5 mm. in diameter and was composed of irregular trabeculae of large hepatic cells and surrounded by flattened liver cells and fibrous tissue.

Rat 340/56 which died 21 months after the last injection had a dark congested liver with a yellow nodule in the left lobe and some haemorrhage into the abdominal cavity. Microscopically all the organs showed much congestion of blood vessels. An area of infarction was present in the liver and a round nodule composed of congested dilated sinusoids with narrow cords of liver cells between them.

Rat 837/56 was killed 25 months after the last injection and the liver showed cystic and yellow areas. Microscopically a multilocular bile duct cyst and a hyperplastic nodule of liver cells were seen.

### *Controls*

Eight male and 7 female rats were kept as controls and received " normal " diet. Liver biopsies were performed on 2 females aged 7 weeks and 3½ months respectively and no architectural abnormalities were found. All control rats survived from 18 to 33 months and showed no cirrhosis, hyperplasia, or tumours of the liver. Seven had extensive bronchiectasis. Three rats had abscesses in uterine horn, jaw, and middle ear respectively. One had a mammary fibroadenoma, and 1 an ovarian tumour with metastases. Three male rats were given " normal" diet supplemented with a 1 per cent solution of betaine instead of drinking water for 1 year. These survived 22–29 months. No abnormalities were seen in their livers.

### DISCUSSION

Rosenberg and Beath (1945) described acute hepatotoxic effects of riddelliine in mice injected intraperitoneally (100–500 mg./kg. body weight), as well as more chronic lesions due to feeding the dried, ground plant of *Senecio riddellii.* In the present experiments riddelliine was tested in rats in which it produced nodular hyperplasia, fibrosis and primary liver tumours. In rats, lesions produced by riddelliine were very similar to those due to other pyrrolizidine alkaloids and developed in animals which appeared in good health during the period of treatment and for many months after the treatment was discontinued. A notable feature was the great variation of the amount of fibrous tissue in livers of individual animals surviving the same time after the treatment with riddelliine and the greater susceptibility of males than females to the action of this alkaloid. The same was observed with the other pyrrolizidine alkaloids, retrorsine and isatidine.

It is well known how difficult it is to diagnose liver tumours and to assess their degree of malignancy, in the absence of metastases (*e.g.* Opie, 1944 ; Magee and Barnes, 1956). Usually authors decide on their own critieria of classification which makes it difficult to compare data from different laboratories. Some more objective critieria are greatly desirable.

The present experiments may serve such a purpose. The treatment was stopped at a time when liver lesions were likely to have developed but were still very slight when examined in biopsy specimens and the animals were allowed to live as long as possible. This procedure showed whether and to what extent the lesions are reversible.

The results show that liver lesions produced by pyrrolizidine alkaloids, retrorsine, riddelliine, and isatidine, are irreversible and progressive. Even in the absence of further treatment the changes present in the livers became more accentuated with time, fibrous tissue if present became more prominent, hyperplastic nodules tended to increase in size, mitotic figures became more numerous while characteristic large parenchymal cells persisted throughout the lifespan of the animals. Although no metastases were seen in the present series of animals some of the liver lesions were distinctly hepatomata. These were found sometimes in livers in which fibrotic changes were not prominent and also in those in which fibrosis was present. Similar lesions have been shown previously to lead to malignant tumours with metastasis (Schoental *et al.*, 1954) and in view of their progressive nature it seems justified to consider these as neoplastic. However the long survival of the animals in apparent good health does not suggest a high degree of malignancy.

The role of nutrition in relation to liver damaging agents in general (Drill, 1952) and to liver carcinogens such as azo-dyes in particular has been extensively studied (Miller and Miller, 1955). Only a few dietary factors have as yet been investigated in conjunction with pyrrolizidine alkaloids. While protein deficiency has been shown to have a deleterious effect on the survival time of the animals treated with pyrrolizidine alkaloids (Selzer, Parker and Sapeika, 1951 ; Schoental and Magee, 1957) in the present pilot experiment high casein diet favoured longer survival of rats and also the development of liver tumours. While choline deficiency is known to produce liver tumours (Copeland and Salmon, 1946), supplementation of normal diet with choline failed to inhibit the development of tumours due to pyrrolizidine alkaloids (Schoental *et al.*, 1954). It seemed of interest to test whether this lack of action of choline was not due to inactivation of the enzyme, choline oxidase, concerned with the oxidation of choline to betaine, an essential step in the mechanism of transmethylation (Dubnoff, 1949). Asano (1955) reported decrease of choline oxidase in liver tumours.

In a pilot experiment the effect of supplementing the " normal " diet with betaine was therefore tested. The results show clearly that betaine, like choline, failed to inhibit, and may have even favoured the development of liver tumours in our animals. It is of interest to note that rats receiving supplements of betaine were not protected against the more acute changes such as anaemia, subcutaneous oedema, gastro-intestinal haemorrhage, pancreatic abnormalities and the corresponding early liver lesions.

The irreversible character of the liver lesions demonstrated by the present experiments illustrates the dangerous potentialities of pyrrolizidine alkaloids when ingested even in doses which may have no immediate effects on the health of the individual. They also underline the obvious difficulty in tracing the causative agents, when confronted with fully developed chronic liver lesions in animals or men.

### SUMMARY

Various liver lesions including liver tumours developed in rats which survived in apparent good health many months after the cessation of a temporary treatment with the pyrrolizidine alkaloids ; riddelliine, retrorsine and isatidine.

Neither high casein diet nor supplements of betaine prevented the development of the various liver lesions.

We wish to thank Dr. P. R. Peacock, the Director of Research, for his interest in this work, Mr. S. Breslin and Mr. C. Bern for the photographs, and the Staff of the Department for their valuable assistance.

For the generous gifts of the alkaloids we are greatly indebted to Professor Roger Adams, University of Illinois, Urbana, (riddelliine), and Professor F. L. Warren, University of Natal, Pietermaritzburg, (retrorsine and isatidine).

### REFERENCES

Adams, R., Hamlin, K. E. Jr., Jelinek, C. F. and Phillips, R. E.—(1942) *J. Amer. chem. Soc.*, **64**, 2760.
Asano, B.—(1955) *Gann*, **46**, 41.
Bruce, H. M. and Parkes, A. S.—(1949) *J. Hyg., Camb.*, **47**, 202.
Copeland, D. H. and Salmon, W. D.—(1946) *Amer. J. Path.*, **22**, 1059.
Cook, J. W., Duffy, E. and Schoental, R.—(1950) *Brit. J. Cancer*, **4**, 405.
Drill, V. A.—(1952) *Pharmacol. Rev.*, **4**, 1.
Dubnoff, J. W.—(1949) *Arch. Biochem.*, **24**, 257.
Magee, P. N. and Barnes, J. M.—(1956) *Brit. J. Cancer*, **10**, 114.
Miller, E. C. and Miller, J. A.—(1955) *J. nat. Cancer Inst.*, **15**, 1571.
Opie, E. L.—(1944) *J. exp. Med.*, **80**, 231.
Rosenberg, I. and Beath, O. A.—(1945) *Amer. J. clin. Path.*, **15**, 407.
Schoental, R.—(1955) *Voeding*, **16**, 268.
*Idem* and Head, M. A.—(1955) *Brit. J. Cancer*, **9**, 229.
*Iidem* and Peacock, P. R.—(1954) *Ibid.*, **8**, 458.
*Idem* and Magee, P. N.—(1957) *J. Path. Bact.*, **74**, 305.
Selzer, G., Parker, R. S. F. and Sapeika, N.—(1951) *Brit. J. exp. Path.*, **32**, 14.